# Chest x-ray feature of venous air embolism in orthopedic surgery in prone position: A case report

**DOI:** 10.3389/fsurg.2022.994839

**Published:** 2023-01-09

**Authors:** Yuwu Liu, Qun Gao, Andi Chen, Jian Xiao, Ping Shi

**Affiliations:** ^1^ Orthopedics Department, Guangyuan Central Hospital, Guangyuan, China; ^2^ Department of Orthopedics, Jiangshan People's Hospital, Jiangshan, China; ^3^ Department of Anesthesiology, Guangyuan Central Hospital, Guangyuan, China; ^4^ Nephrology Department, Guangyuan Central Hospital, Guangyuan, China; ^5^ Department of Respiratory Medicine, Guangyuan Central Hospital, Guangyuan, China

**Keywords:** chest x-ray, venous air embolism, prone position, esophageal ultrasound, case report

## Abstract

**Background:**

Venous air embolism (VAE) is a life-threatening event characterized as a series of clinical features of the disease caused by gas entering the venous circulation in the body.

**Case presentation:**

A 72-year-old male patient with an ankle fracture after trauma was admitted, and complained of chest pain and dyspnea after the ankle fracture resection and internal fixation. His heart rate and blood pressure dropped, and the patient was diagnosed with VAE according to a chest x-ray and clinical features. Cardiopulmonary resuscitation was carried out and the patient's heartbeat recovered; his blood pressure rose to a normal level. The patient was still unconscious and sent to the intensive care unit for continued monitoring and treatment. Unfortunately, the patient discharged himself from the hospital and died 24 h later.

**Conclusion:**

This case suggests that x-ray may be a potential method for the rapid diagnosis of VAE in a resource-limited setting.

## Introduction

Air embolism is a fatal event in surgery. Venous air embolism (VAE) is well known in neuroanesthesia, with established surgeries and sites where its incidence is high. Therefore, surgeries and positions are notorious for the incidence of VAE, such as posterior cervical spine surgeries, sitting craniotomies, infratentorial surgeries, and craniosynostosis repairs. However, VAE may also affect patients in the prone position (1). The clinical feature caused by VAE is similar to the clinical feature of pulmonary embolism caused by venous thromboembolism. It is difficult to quickly diagnose VAE based on the clinical feature alone. Esophageal ultrasound, Doppler ultrasound, ETCO_2_, and CT have their advantages, but there are also many shortcomings. This case proposes chest x-ray as a better method for the rapid diagnosis of fatal VAE.

## Case presentation

**Figure 1:**
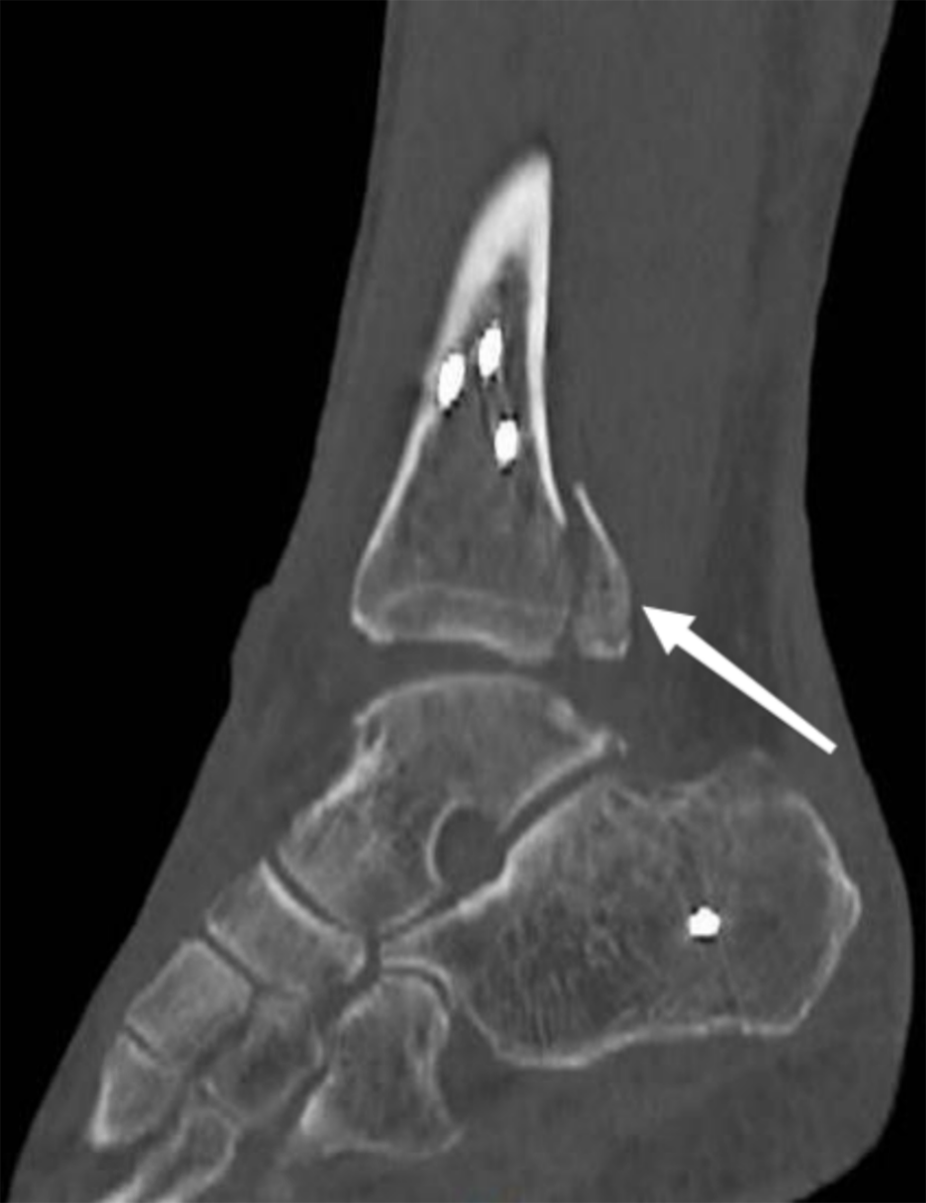
Ankle fracture after trauma.

**Figure 2:**
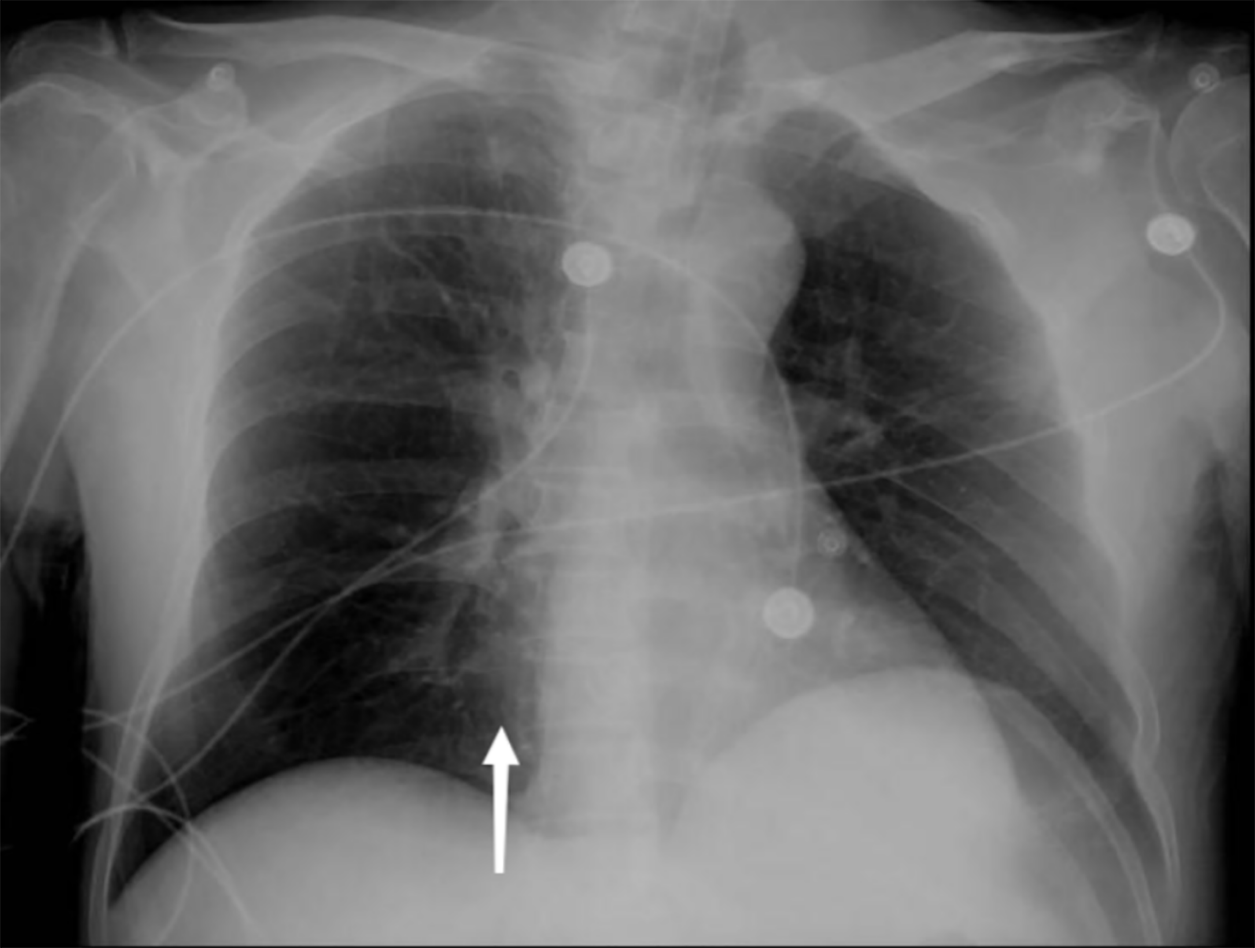
The patient's pulmonary visual field brightness increased, and the right ventricular margin was extremely dilated. The right ventricular lucency increased, and there was a honeycomb-like change in the right ventricle. The pulmonary artery was occupied by air, and the pulmonary artery margin was dilated, and the pulmonary artery lucency significantly increased.

A 72-year-old male patient was admitted to the orthopedic department of our hospital for an ankle fracture after a traffic accident injury in May 2021, without any related medical history. He underwent an open reduction and internal fixation for a fracture of the medial lateral and posterior ankle diagnosed by x-ray (Figure 1). Because it was a posterior ankle fracture, the patient was placed in a prone position and a posterolateral approach incision from the ankle was made. Approximately 1.5 h after the operation, the patient suddenly complained of chest pain and dyspnea. He was assisted in turning over to the supine position and was given oxygen immediately; venous access was established by a central venous catheter (CVC). The patient's dyspnea did not improve, and his blood pressure dropped rapidly. His heartbeat stopped and cardiopulmonary resuscitation (CPR) was performed. A pulmonary embolism caused by a blood clot was first considered for the typical presentation of dyspnea, decreased blood pressure, chest pain, and oxygen saturation, and 1.5×10^6^ U urokinase was administrated. The patient's heartbeat did not recover. During a pause in performing CPR, we decided to take a chest x-ray and found the right heart and pulmonary artery full of air; the increase in the light transmittance of the visual field in both lungs was consistent with the characteristics of a pulmonary embolism (Figure 2). Air was also drawn from the CVC; VAE was then considered based on the imaging results, and the patient was treated conservatively by positioning him head down and tilted to the left (Durant's position) (2) The CPR was continued. The patient's heartbeat recovered, and his blood pressure rose to a normal level. He was still unconscious and sent to the intensive care unit. Unfortunately, the patient was discharged from the hospital and died 24 h later. We suspect the patient died due to anoxia or heart-related complications. However, no autopsy was performed.

## Discussion

In this case, a 72-year-old patient with an ankle fracture after a traffic accident injury underwent an ankle fracture open reduction and internal fixation. He complained of chest pain and dyspnea, and was diagnosed as having a VAE according to x-ray results in the prone position, which is an intuitive, convenient, and widely used method.

Some studies have shown that the prone surgical position can cause VAE (3–9). During prone surgery, the distance of the entrance point above the right side of the heart is increased and can generate gravity gradients even without blood loss (10). This gravity gradient allows peripheral air to enter the venous circulation, then moving to the right ventricle and pulmonary artery, causing symptoms of pulmonary embolism (7, 10, 11). Because of the surgical position, neurosurgeons are more familiar with pulmonary embolism caused by VAE. However, few orthopedic surgeons know that the prone position can lead to a VAE by causing air to enter the venous access and progress to a pulmonary embolism. However few orthopedic surgeons aware that the prone position can lead to air entering the venous access and developing a pulmonary embolism. Furthermore, fatal VAEs occur very rarely and are poorly understood by orthopedic surgeons. In addition, a fatal VAE can cause chest pain, cyanosis, dyspnea, a drop in blood pressure, increased pulmonary artery pressure, cardiac arrest, and other clinical features (7, 10, 12, 13). These symptoms are similar to those of a pulmonary embolism caused by deep vein thrombosis, which was confused by orthopedic surgeons. A VAE during orthopedic surgery is often misdiagnosed as thrombosis. Therefore, when a fatal VAE occurs, it is important to obtain a clear diagnosis early and quickly to save the patient's life (13). To quickly diagnose a pulmonary embolism caused by a VAE, it is necessary to enhance the orthopedic surgeon’s knowledge of VAE and recognize that some surgical positions, including the sitting, prone, and lateral positions, may lead to the risk of a VAE. Orthopedics also need to work closely with anesthesiologists and surgical nurses. Once symptoms of a pulmonary embolism appeared, the infusion pipeline and deep venous catheterization should be checked immediately to observe whether gas has entered.

Convenient medical equipment is needed for further diagnosis. A CT examination of a VAE can obtain relatively intuitive images. However, it is almost impossible to perform CT examinations on patients while they are undergoing CPR. Transesophageal echocardiography (TOE) and Doppler ultrasound are top sensitive examination equipment, but they are expensive and invasive (10, 14). Moreover, the inspection requires an experienced operator, which limits its use (15, 16). Monitoring ETCO_2_ changes is a practical measure. However, this is a less sensitive and nonspecific feature and lacks direct imaging evidence (4, 16). A typical clinical sign of a VAE is that a “mill-wheel”-like murmur can be heard during auscultation, but this kind of mill-wheel noise is not always heard (4, 10, 17).

A pulmonary embolism caused by a gas embolism can be seen on x-ray chest radiographs as insufficient lung tissue circulation. The increase in the light transmittance of the visual field in both lungs in this case is consistent with the characteristics of a pulmonary embolism. When the gas occupies the right ventricle, the edge of the right ventricle can be found to expand, and the translucency of the right ventricle increases (18, 19). The honeycomb-like changes in the right ventricle can be seen. After the pulmonary artery is occupied by gas, the edge of the pulmonary artery can be seen to expand, and the pulmonary artery transmittance is significantly increased (19). This is the main feature of a large amount of gas embolism. X-ray equipment is convenient medical equipment, which was widely used in resource-limited settings. For patients suspected of having a fatal VAE during surgery, chest imaging can be performed during the patient's cardiopulmonary resuscitation, and the results of the imaging can be obtained quickly, which can be early distinguished VAE from VTE. The identification of an embolism and obtaining a clear diagnosis of a VAE are vital to saving the lives of patients.

In conclusion, orthopedics needs pay more attention to VAEs and x-ray equipment is a convenient, widely used medical device that can provide a rapid diagnosis for a fatal VAE.

## Data Availability

The original contributions presented in the study are included in the article/Supplementary Material, further inquiries can be directed to the corresponding author.

## References

[1] Vinay B Sriganesh K Krishna KNG. An abrupt reduction in end-tidal carbon-dioxide during neurosurgery is not always due to venous air embolism: a capnograph artefact. J Clin Monit Comput. (2014) 28(2):217–9. 10.1007/s10877-013-9505-y 23996497

[2] Yeung E Adeboye A Granet P Casos S. Rare pathology in a trauma patient: air embolism following peripheral intravenous access. BMJ Case Rep. (2021) 14(1):e240428. 10.1136/bcr-2020-240428 33509893 PMC7845703

[3] Sutherland R Winter R. Two cases of fatal air embolism in children undergoing scoliosis surgery. Acta Anaesthesiol Scand. (1997) 41(8):1073–6. 10.1111/j.1399-6576.1997.tb04839.x 9311410

[4] Albin MS Ritter RR Pruett CE Kalff K. Venous air embolism during lumbar laminectomy in the prone position: report of three cases. Anesth Analg. (1991) 73(3):346–9. 10.1213/00000539-199109000-00021 1867429

[5] Lopez L Traves N Napal M. Fatal gas embolism during corrective surgery for scoliosis using the posterior approach. Rev Esp Anestesiol Reanim. (1999) 46(6):267–70. Spanish. PMID: 10439648

[6] Horlocker TT Wedel DJ Cucchiara RF. Venous air embolism during spinal instrumentation and fusion in the prone position. Anesth Analg. (1992) 75(1):152–00; author reply 153. 10.1213/00000539-199207000-00045 1616150

[7] Wills J Schwend RM Paterson A Albin MS. Intraoperative visible bubbling of air may be the first sign of venous air embolism during posterior surgery for scoliosis. Spine. (2005) 30(20):E629–35. 10.1097/01.brs.0000182347.85827.0c 16227882

[8] Shenkin HN Goldfedder P. Air embolism from exposure of posterior cranial fossa in prone position. JAMA. (1969) 210(4):726. 10.1001/jama.1969.03160300066022 5394410

[9] Elouardi Y Zarrouki Y Darouich H Khallouki M. Fatal venous air embolism during lumbar spondylolisthesis surgery. Indian J Anaesth. (2021) 65(2):171. 10.4103/ija.IJA_575_20 33776100 PMC7983818

[10] Albin MS Carroll RG Maroon JC. Clinical considerations concerning detection of venous air embolism. Neurosurgery. (1978) 3(3):380–4. 10.1227/00006123-197811000-00009 740137

[11] Distefano VJ Klein KS Nixon JE Andrews ET. Intra-operative analysis of the effects of position and body habitus on surgery of the low back: a preliminary report. Clin Orthop Relat Res. (1974) 99:51–6. 10.1097/00003086-197403000-00005 4825719

[12] Bothma PA Schlimp CJ. II. Retrograde cerebral venous gas embolism: are we missing too many cases? Br J Anaesth. (2014) 112(3):401–4. 10.1093/bja/aet433 24355833

[13] Guo J-L Wang H-B Wang H Le Y He J Zheng X-Q Transesophageal echocardiography detection of air embolism during endoscopic surgery and validity of hyperbaric oxygen therapy: case report. Medicine (Baltimore). (2021) 100(23):e26304. 10.1097/MD.0000000000026304 34115039 PMC8202586

[14] Albin MS. Venous air embolism: a warning not to be complacent—we should listen to the drumbeat of history. Anesthesiology. (2011) 115(3):626–9. 10.1097/ALN.0b013e31822a6408 21799396

[15] Lee YL Hwang KY Yew WS Ng SY. An abnormal capnography trace due to air embolism in the lateral position. BMJ Case Reports CP. (2019) 12(8):e231316. 10.1136/bcr-2019-231316 PMC6721413 31466962

[16] Mirski MA Lele AV Fitzsimmons L Toung TJ Warltier DC. Diagnosis and treatment of vascular air embolism. J Am Soc Anesthesiol. (2007) 106(1):164–77. 10.1097/00000542-200701000-00026 17197859

[17] Albin MS Warner DS. Venous air embolism: a warning not to be complacent—we should listen to the drumbeat of history. J Am Soc Anesthesiol. (2011) 115(3):626–9. 10.1097/ALN.0b013e31822a6408 21799396

[18] Toung TJ Rossberg MI Hutchins GM. Volume of air in a lethal venous air embolism. Anesthesiology. (2001) 94(2):360–1. Erratum in: *Anesthesiology* 2001;**94**(4):723. 10.1097/00000542-200102000-00031 11176104

[19] Senn N. An experimental and clinical study of air-embolism. Ann Surg. (1885) 1(6):517–49. 10.1097/00000658-188501000-00002 17855995 PMC1431330

